# Comparative genotyping of SARS-CoV-2 among Egyptian patients: near-full length genomic sequences versus selected spike and nucleocapsid regions

**DOI:** 10.1007/s00430-023-00783-8

**Published:** 2023-10-04

**Authors:** Rasha Emad, Iman S. Naga

**Affiliations:** 1 Alexandria Main University Hospital, Alexandria, Egypt; 2 Department of Microbiology, Medical Research Institute, Alexandria University, Alexandria, Egypt

**Keywords:** GISAID, Nucleocapsid, Nextclade, Spike, SARS-CoV-2, Whole genome sequencing

## Abstract

Several tools have been developed for severe acute respiratory syndrome coronavirus-2 (SARS-CoV-2) genotyping based on either whole genome or spike sequencing. We aimed to highlight the molecular epidemiological landscape of SARS-CoV-2 in Egypt since the start of the pandemic, to describe discrepancies between the 3 typing tools: Global Initiative on Sharing Avian Influenza Data (GISAID), Nextclade, and Phylogenetic Assignment of Named Global Outbreak Lineages (PANGOLIN) and to assess the fitness of spike and nucleocapsid regions for lineage assignment compared to the whole genome. A total of 3935 sequences isolated from Egypt (March 2020–2023) were retrieved from the GISAID database. A subset of data (n = 1212) with high coverage whole genome was used for tool discrimination and agreement analyses. Among 1212 sequences, the highest discriminatory power was 0.895 for PANGOLIN, followed by GISAID (0.872) and Nextclade (0.866). There was a statistically significant difference (p = 0.0418) between lineages assigned via spike (30%) and nucleocapsid (46%) compared to their whole genome-assigned lineages. The first 3 pandemic waves were dominated by B.1, followed by C.36 and then C.36.3, while the fourth to sixth waves were dominated by the B.1.617.2, BA, and BA.5.2 lineages, respectively. Current shift in lineage typing to recombinant forms. The 3 typing tools showed comparable discrimination among SARS-CoV-2 lineages. The nucleocapsid region could be used for lineage assignment.

## Introduction

In December 2019, life-threatening viral pneumonia was reported in Wuhan, Hubei, China, leading to a high fatality rate in China that then became a worldwide pandemic [1]. This viral pneumonia was called coronavirus disease 2019 (COVID-19), which is caused by a novel coronavirus named severe acute respiratory syndrome coronavirus 2 (SARS-CoV-2) [2]. In Egypt, the first (index) case confirmed with SARS-CoV-2 viral infection was reported on 14 February 2020 in Cairo [3]. Egypt was the first African country to announce the presence of COVID-19. A month later (in mid-March), the first two whole genome sequences of SARS-CoV-2 isolated from Egyptian citizens were published [4].

On 11 March 2020, the World Health Organization (WHO) announced that the COVID-19 outbreak was a global pandemic. Since the start of the pandemic, Egypt has been ranked one of the top 5 countries reporting COVID-19 cases in Africa [5].

There are four phylogenetic groups of coronaviruses known as alpha, beta, gamma, and delta. SARS-CoV-2 is a beta-coronavirus that is further divided into 4 main lineages (A–D) [6]. As SARS-CoV-2 is highly prone to multiple recombination events, mutations are introduced in its genome that could cause changes in antiviral susceptibility and viral transmission, resulting in the emergence of new recombinant variants [7].

According to the SARS-CoV-2 Interagency Group (Updated March. 20, 2023), there are 4 types of variants: variant of interest (VOI), variant of concern (VOC), variant of high consequence (VOHC), and variants being monitored (VBM). This classification is dynamic and always updated according to the circulating lineages. Currently, there are no SARS-CoV-2 variants described as VOI or VOHC [8]. Another classification, the PANGO lineage system, depends on hierarchical evolution from a parent lineage. It consists of an alphabetical prefix and numerical suffix describing this hierarchy.

To avoid long names for lineages, another alphabetical synonymous is given such as “BA” stands for “B.1.1.529” (omicron variant) or “AY” stands for “B.1.617.2” (delta variant). For a comprehensive list of abbreviations for lineage long names, refer to the following link https://github.com/cov-lineages/pangodesignation/blob/master/pango_designation/alias_key.json. The first omicron variant detected in Egypt was reported by Ismail et al. [9] on 9 December 2021 with the accession number EPI_ISL_7952324.

Three well-known databases or software tools exist to track SARS-CoV-2 molecular evolution by analyzing the genomic sequences to determine clade, lineage, variant and mutations: Global Initiative on Sharing Avian Influenza Data (GISAID, v2.5.1) [https://gisaid.org/] [10–12], Nextclade v2.14.1 [https://clades.nextstrain.org] [13] (a part of Nextstrain), and Phylogenetic Assignment of Named Global Outbreak Lineages (PANGOLIN, v4.3, data version v1.20) [https://pangolin.cog-uk.io/] [14, 15].

In Egypt, from 3 January 2020 to 24 May 2023, there have been 516,023 confirmed cases of COVID-19 with 24,830 deaths reported to the WHO. Despite the recent announcement of the end of the pandemic on 5 May 2023, a relatively large number of SARS-CoV-2 sequences are still being submitted to GISAID.

In this work, we aimed to highlight the molecular epidemiological landscape of SARS-CoV-2 over the time since the first appearance of the pandemic in Egypt in February 2020. Furthermore, we aimed to describe discrepancies between the 3 well-known lineage assigner software tools (GISAID, Nextclade, and PANGOLIN) for SARS-CoV-2 typing and to determine their agreement according to the assigned lineage. Finally, we assessed the fitness of selected regions, such as spike (S) and nucleocapsid (N), compared to whole genome sequences for lineage typing.

## Methods

### Data collection

**Fig. 1:**
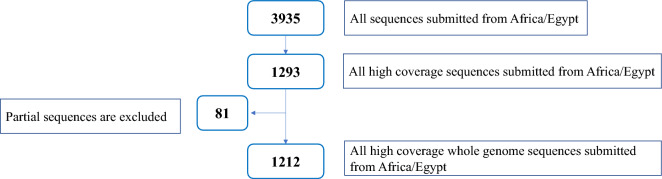
Flowchart demonstrating inclusion criteria for the sequences according to aims of the study

A total of 3935 sequences of SARS-CoV-2 isolated from Egypt with their associated metadata, including clade, lineage, and variant, were retrieved from the GISAID database [11] in April 2023 after applying the following filters: location: Africa/Egypt, and host: human. These sequences were either complete genome “ > 29,000 bp” or partial genomic regions such as spike or nucleocapsid, which were all submitted across different governates of Egypt since the start of the pandemic in early 2020. A subset of data consisting of high-coverage complete genomic sequences (n = 1212) was utilized for lineage discrimination, and low-quality and/or partial sequences were excluded from this analysis. We used a GISAID high coverage filter for the exclusion of low-quality sequences (Fig. 1). After that, we manually excluded a group of partial sequences (n = 81). A high coverage sequence is defined as a sequence with less than 5% ambiguous bases (NNNs) and no deletions or insertions without the verification of the submitter according to GISAID.

The 1212 sequences with a whole genome determined by GISAID were aligned against the sequence of the reference genome NC_045512.2 in BioEdit software [16] using the ClustalW multiple alignment tool. We extracted the sequences of the spike (S) region (3822 bp, 21,563–25,384) and nucleocapsid (N) region (1260 bp, 28,274–29,533) from the whole genome sequences for further analysis.

### Genotyping and data analysis

The whole genome of the whole dataset (n = 3935) was subjected to genotyping by Nextclade and PANGOLIN online tools, while the genotyping data by GISAID were already available within downloaded metadata. After that, we visualized the top 5 lineage distributions per wave in Egypt. The smaller subset of data (n = 1212) was subjected to sequence extraction of the S and N regions and further genotyping by the Nextclade tool.

Two approaches for analysis were utilized. First, we compared agreement on lineage assignment of whole genome sequences between the 3 online freely available tools GISAID, PANGOLIN and Nextclade to detect discrepancies. After that, we investigated the agreement of lineage assignment between the S and N regions compared to the whole genome.

### Data snapshot

The findings of this study are based on metadata associated with two datasets. The first dataset “EPI_SET_230521kt” (accessible at https://doi.org/10.55876/gis8.230521kt) is composed of 3935 individual genome sequences. The collection dates range from 13 March 2020 to 12 March 2023, while the second dataset “EPI_SET_230415te” (accessible at https://doi.org/10.55876/gis8.230415te) is composed of 1212 individual high-coverage whole genome sequences. The collection dates ranged from 9 March 2020 to 13 March 2023. Data were collected from Africa/Egypt. All sequences in these two datasets were compared relative to hCoV-19/Wuhan/WIV04/2019 (WIV04), the official reference sequence employed by GISAID (EPI_ISL_402124).

### Statistical methods

We used the discriminatory power (D) (https://insilico.ehu.es/mini_tools/discriminatory_power/index.php) as a numerical index for the discrimination power between lineage assigner software tools. where N is the total number of isolates in the typing method, s is the number of distinct patterns discriminated by the tool, and nj is the number of isolates belonging to the *j*^th^ pattern [17, 18].

$$D=1-\frac{1}{N \left(N-1\right)}\sum_{j=1}^{S}nj (nj-1)$$

The agreement analysis was performed using the receiver operating characteristic curve (ROC) and area under the curve (AUC) method. We compared the categorical variables by the chi-square test. The alpha level was set at ≤ 0.05. We performed all statistical tests in Rstudio (R 4.2.3) [19]. The pROC package [20] was used to generate the ROC/AUC curves.

### Phylogenetic and analysis

We created a phylogenetic tree by MAFFT [21] version 7.475, TrimAl [22] version v1.4.rev15, and IQ-TREE [23] version 2.0.3. The tree model was GTR + F + R3 with the refseq trim method. The tree was generated by the online phylogenetic tool [24–27] (https://ngdc.cncb.ac.cn/ncov/online/tool/tree). The tree was subjected to scalable clock phylogenetic dating by treedater package in RStudio [28]. After that, the tree was exported as a Newick file, visualized by iTOL [29, 30] and annotated by the iTOL annotation editor (https://itoleditor.letunic.com).

## Results

### Description of circulating lineages

The majority of Egyptian sequences included in the current research were submitted to the GISAID database in 2022 (1707/3935, 43.4%) followed by 2021 (1170/3935, 29.7%). The highest number of sequences submitted was in the fourth quarter in 2021 (N = 567) and the first quarter in 2022 (N = 678).

According to GISAID, the 3935 sequences were grouped into 10 clades as follows: GRA (1614/3935), GR (792/3935), GK (653/3935), GH (393/3935), G (270/3935), O (133/3935), L (32/3935), S (26/3935), GRY (17/3935), and GV (5/3935).

The ability of the 3 tools to assign a lineage was different. Among the whole dataset (n = 3935), a total of 427 sequences were unassigned by GISAID either due to being partial sequences or low-quality whole genome sequences. There were 516 sequences unassigned by PANGOLIN due to the inability of the tool to process the sequence. On the other hand, Nextclade was able to assign almost the whole dataset except 10 sequences.

Among all sequences (n = 3935), during the first wave, the most frequent lineage was B.1 (312 sequences), followed by C.36 (123 sequences). In the second wave, the situation was reversed, and C.36 (221 sequences) became the most common lineage, followed by B.1 (79 sequences). Despite the small number of submitted sequences during the third wave, C.36.3 (74 sequences) was the most frequent lineage, followed by C.36 (42 sequences).

In the fourth wave, the count of detected lineages was higher than in previous waves, indicating higher diversity among circulating lineages. A majority of sequences (n = 277) were unassigned, while the most frequent lineage was B.1.617.2 (140 sequences), followed by AY.122 (117 sequences). On the other hand, during the fifth wave, BA.1 (71 sequences) and BA.2 (79 sequences) became dominant compared to the previous circulating lineages. In the sixth wave, BA.5.2 (329 sequences) was the utmost circulating lineage, followed by BA.2 (145 sequences).

**Fig. 2:**
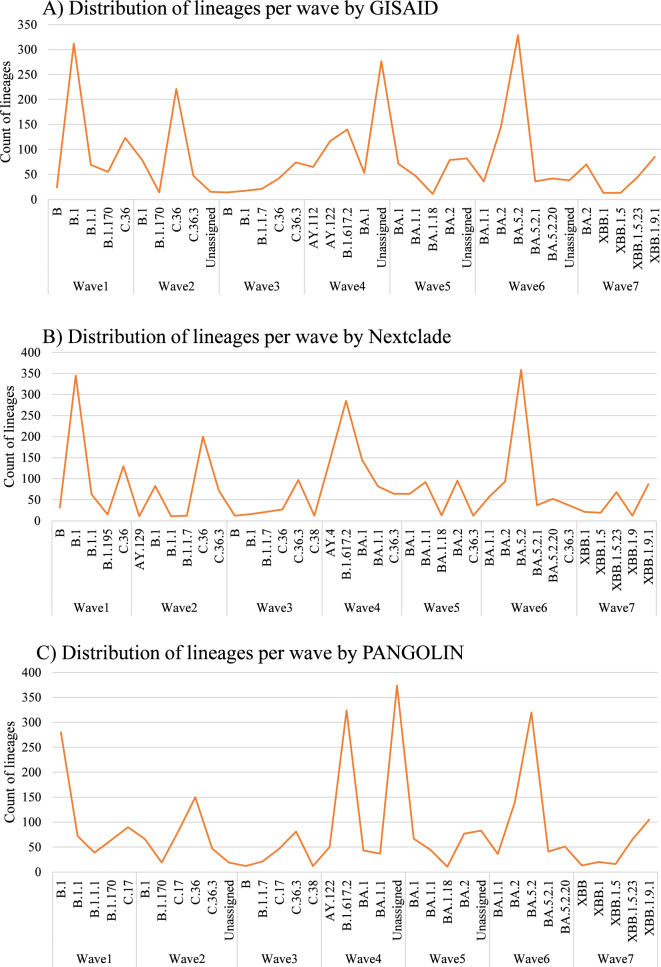
Distribution of the top 5 SARS-CoV-2 lineages per wave according to 3 lineage assigner tools. **A** GISAID, **B** Nextclade, and **C** PANGOLIN among the whole dataset (n = 3935) in Egypt. Figure was generated by Microsoft Excel version 2021

Lastly, in wave 7, it is noteworthy that starting from late 2022 (Q4) and early 2023 (Q1), the recombinant lineages (XBB) became predominant compared to other nonrecombinant lineages. Figure 2 illustrates the distribution of lineages according to GISAID, Nextclade, and PANGOLIN since the start of the pandemic and across the wave pattern in Egypt.

### Discrimination between typing tools

Among the 1212 high-coverage whole genome sequences, the discriminatory power of the GISAID tool was 0.872 for differentiation between 72 lineages, while the discriminatory power of the Nextclade tool was 0.866 for differentiation between 62 lineages. Finally, the discriminatory power of PANGOLIN was 0.895 for differentiation between 57 lineages.

### Agreement analysis

#### Overall agreement between tools

The 3 tools GISAID, PANGOLIN and Nextclade showed overall agreement with exact matches in 744/1212 (61.4%) of the whole genome sequences, while the remaining 468 (38.6%) sequences showed variable discrepancies among the 3 tools. Finally, the overall disagreement between the 3 lineage assigner tools was estimated to be 76/1212 (6.3%) (Table 1).

**Table 1:** Pairwise, overall agreement and disagreement level between the 3 lineage assigner tools

Agreement between tools N = 1212	GISAID	Nextclade	Overall agreement	Overall disagreement
Nextclade	855 (70.5%)	1212 (100%)	744 (61.4%)	76 (6.3%)
PANGOLIN	885 (73%)	876 (72.3%)		

*GISAID* global initiative on sharing all influenza data

*PANGOLIN* phylogenetic assignment of named global outbreak lineages

#### ROC and AUC analysis for each lineage

**Fig. 3:**
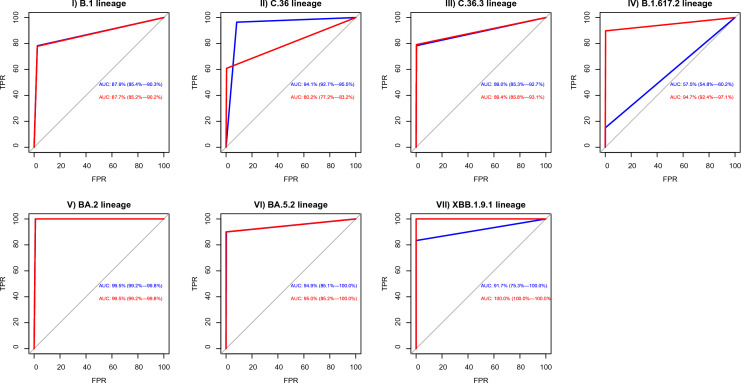
Receiver operating characteristic/area under the curve (ROC/AUC) curves for lineage assignment agreement between Nextclade (reference typing method), GISAID, and PANGOLIN for 7 main lineages, including B.1, C.36, C.36.3, and B.1.617.2. BA.2, BA.5.2, and XBB.1.9.1. The BLUE line represents the GISAID tool, and the RED line represents the PANGOLIN tool. *TPR* true positive rate, *FPR* false positive rate, *AUC* area under the curve

We conducted a ROC and AUC method to demonstrate agreement between tools for 7 main lineages (Fig. 3). We observed that 3 tools agreed with > 85% AUC on 6 lineages: B.1, C.36, C.36.3, BA.2, BA.5.2, and XBB.1.9.1. In the case of lineage B.1.617.2, the GISAID tool showed a poor AUC (57.5%) compared to PANGOLIN (94.7%).

#### Agreement between regions

After S and N region extraction from the 1212 whole genome sequences, we compared the S and N lineage assignments to their corresponding assignments by Nextclade whole genome. There was a statistically significant difference (p = 0.0418*) between the agreement of the S region and N region with the whole genome lineage by Nextclade (Table 2). We observed that the N region agrees with whole genome with a higher percentage (46%) compared to the S region (29.9%). Hence, we concluded that the N region could be utilized as an alternative to the S region in lineage assignment for SARS-CoV-2 sequences.

**Table 2:** Comparison between spike and nucleocapsid agreement with the whole genome in lineage assignment according to Nextclade among 1212 high-coverage whole genome sequences

Region name	Nucleocapsid		Total	X^2^	P value
Spike	Agree with WG	Disagree with WG			
Agree with WG	150 (12.4%)	212 (17.5)	362 (30%)	4.14	0.0418*
Disagree with WG	408 (33.6%)	442 (36.5%)	850 (70.%)		
Total	558 (46%)	654 (54%)	1212 (100%)		

The bold p value indicates a significant difference between the agreement of the S and N regions to the whole genome at an alpha level < 0.05

*WG* whole genome sequence, *X*^*2*^ Chi-square test

### Phylogenetic and clustering analyses

**Fig. 4:**
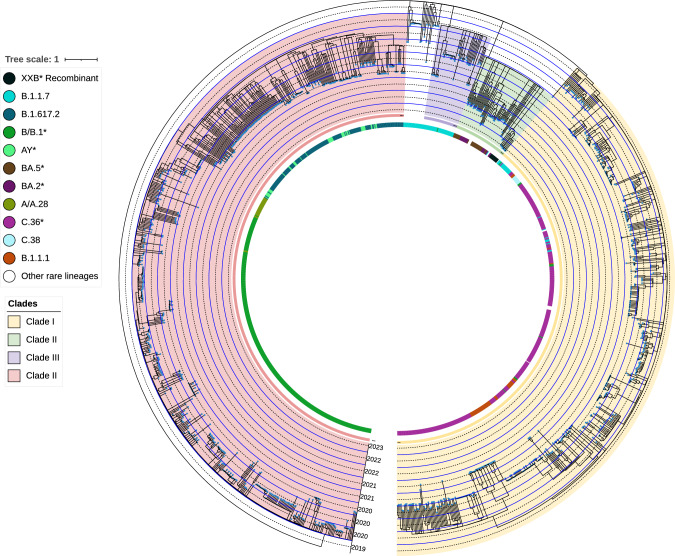
Time-resolved phylogenetic tree created by MAFFT version 7.475, TrimAl version v1.4.rev15, IQ-TREE version 2.0.3 and treedater in RStudio. The tree model was GTR + F + R3 with the refseq trim method. This tree highlights the 1212 high-coverage whole genome sequences included in the study. Concentric circles represent sampling years. Each tip represents a single sample. The tree was visualized by iTOL and annotated by the iTOL annotation editor [29]. * indicates all sublineages. Two large and 2 small clades are colored. Colored strip represents circulating pango lineage according to Nextclade

Two large clusters were visualized, one encompassing a total of 607 sequences, including mainly C.36, C.36.3, BA.2, and BA.5 lineages, while the other cluster contained a total of 599 sequences, including mainly B.1, B.1.617.2 (parent delta variant), and constellation of AY* sublineages (Fig. 4).

## Discussion

Globally, as of 25 May 2023, 15,608,522 SARS-CoV-2 genomic sequences have been submitted to GISAID. In the current work, as of 10 April 2023 (date of access), a total of 3935 SARS-CoV-2 genomic sequences submitted by Egypt since the start of the pandemic and over 3 years (March 2020–March 2023) were downloaded from the GISAID database. We aimed to highlight the shift in lineage assignment across wave patterns in Egypt.

The coronavirus genome averages approximately 29 (26–32) kb, which is identified as the largest genome size for an RNA virus [31]. Indeed, whole genome sequencing is the best option for lineage assignment; however, few drawbacks, such as high cost and time consumption, exist in low-resource countries such as Egypt. Therefore, sequencing of smaller regions instead of the whole genome is considered more feasible under these circumstances.

According to the official site of the Egyptian Ministry of Health (https://www.care.gov.eg), the first pandemic wave in Egypt was from April 2020 to September 2020, while the second pandemic wave was from October 2020 to March 2020. The peaks of the first and second waves of COVID-19 in Egypt were in mid-June and late December 2020, respectively. The first wave was dominated by B variants, especially B.1, similar to other parts in the world at that time. During the following second and third waves, a shift in lineage was observed in the C.36 and C.36.3 lineages. Then, the delta variant (B.1.617.2) and omicron variant (B.1.1.529) became dominant during the fourth and fifth waves. Interestingly, there was a current shift in prevalence of circulating lineages from dominant nonrecombinant forms such as B.1 and C.36.3 to recombinant forms such as XBB.1.9.1. These recombinant forms were circulating at low levels during the first year of the pandemic [32]

Several software tools have been developed specifically for SARS-CoV-2 genotyping based on whole genome and/or partial domain sequencing, such as GISAID, PANGOLIN, and Nextclade. According to the European Centre for Disease Prevention and Control (ECDC), whole genome sequencing (WGS), or at least complete or partial S region sequencing, is the best method for assigning a specific lineage or variant [33].

A study addressing the genomic diversity of SARS-CoV-2 among North African countries, including Egypt, was conducted in December 2021 [34]. They analyzed a total of 1669 whole genome sequences, of which 971 high-coverage sequences were from Egypt. They reported the distribution of lineages as C.36 (30.6%), followed by B.1 (25.2%), C.36.3 (7.2%), B.1.1 and B.1.617.2, with 5.1% each according to the PANGOLIN tool.

A previous Egyptian study reported a shift in lineage prevalence from B.1 to B.1.1.1 between wave 1 and wave 2 [35]. However, we observed a shift in lineage from B.1 to C.36 between wave 1 and wave 2 in our study. This disagreement may be attributed to the current analysis being performed after the end of pandemic waves. According to GISAID, the C.36 lineage was detected early during the pandemic (in May2020) in Egypt and continued to circulate within the country at variable levels.

In this study, we aimed to evaluate the discriminatory power of each tool. All 3 tools showed comparable discriminatory power: GISAID (0.872), PANGOLIN (0.895), and Nextclade (0.866). Because the 3 software tools exhibit different nomenclature and classification systems for lineage assignment, discrepancies between tools were expected.

Here, we can demonstrate one particular discrepancy due to the different nomenclature systems. Among 1212 sequences, AY* sublineages were detected in 184, 44 and 58 sequences according to GISAID, Nextclade, and PANGOLIN, respectively. On the other hand, the B.1.617.2 lineage (parent lineage of AY*) was detected in 26, 166, and 152 sequences according to GISAID, Nextclade, and PANGOLIN, respectively. This may be explained by the improved ability of GISAID to classify sublineages to AY* rather than their parent lineage B.1.617.2. We confirmed this theory by ROC/AUC curves. All 3 tools showed high agreement with AUC > 85%, except in the case of lineage B.1.617.2, and the GISAID tool showed a poor AUC (57.5%) compared to PANGOLIN (94.7%).

Here, we conducted comparative analyses of COVID-19 genotyping derived based on the nucleocapsid region (28,274–29,533 in the NC_045512.2 reference genome) and spike region (21,563–25,384 in the NC_045512.2 reference genome) extracted from high-coverage whole genome sequences of 1212 COVID-19-infected patients from Egypt and submitted to the GISAID EpiCov database since the start of the pandemic.

In this study, we selected the Nextclade tool as the reference typing method for several reasons; it has a high ability to assign lineages (3925/3935, 99.7%) and hence can assign the majority of partial or low coverage sequences that were unassigned by other tools. Nextclade was able to assign almost the whole dataset except for 10 sequences.

Despite the presence of some discrepancies in lineage assignment between the tools, all 3 agreed on assigning the most common lineage circulating per wave during the pandemic in Egypt. B1 was the most common in wave 1, C.36 was most common in wave 2, C.36.3 was most common in wave 3, B.1.617.2 was most frequent in wave 4, BA.2 was most frequent in wave 5, BA.5.2 was the most frequent in wave 6, and recombinant forms (particularly XBB.1.9.1) became predominant (Fig. 2).

We proposed that the N gene may be superior in lineage assignment compared to the S gene. A statistically significant difference (p = 0.04) was observed between S and N agreement with the whole genome, suggesting that the N region agrees with the whole genome more than the S region. Despite, the higher agreement of N region (46%) with whole genome compared to spike agreement (30%), both regions are maybe less sufficient than whole genome which is the best for lineage determination. To the best of our knowledge, this work is the first to explore the ability of another region other than the spike protein for rapid lineage assignment for SARS-CoV-2 sequences.

## Conclusions

There is a current evident shift in lineage assignment toward recombinant forms (XBB), particularly XBB.1.9.1. The 3 lineage assigner tools (GISAID, Nextclade, and PANGOLIN) showed comparable discrimination among the SARS-CoV-2 lineages. We concluded that the N region could be utilized for lineage assignment upon comparing versus spike. However, it is more important to obtain S sequences rather than N sequences since the mutations acquired in S gene are most relevant to escape from the neutralising antibodies. Therefore, for epidimological aspects, it is most important to monitor S gene to be able to identify newly emerging variants.

## Data Availability

The GISAID identifier is EPI_SET_230521kt for the first dataset, while the GISAID identifier for the second dataset is EPI_SET_230415te. All genome sequences and associated metadata in this research are published in GISAID’s EpiCoV database. To view the contributors of each individual sequence with details such as accession number, virus name, collection date, originating lab and submitting lab and the list of Authors, visit the following https://doi.org/10.55876/gis8.230521kt for the first dataset (n = 3935) and https://doi.org/10.55876/gis8.230415te for the second dataset (n = 1212).
